# Impact of retrospective data verification to prepare the ICON6 trial for use in a marketing authorization application

**DOI:** 10.1177/1740774519862528

**Published:** 2019-07-26

**Authors:** Andrew Embleton-Thirsk, Elizabeth Deane, Stephen Townsend, Laura Farrelly, Babasola Popoola, Judith Parker, Gordon Rustin, Matthew Sydes, Mahesh Parmar, Jonathan Ledermann, Richard Kaplan

**Affiliations:** 1 MRC Clinical Trials Unit, University College London, London, UK; 2 Comprehensive Clinical Trials Unit, University College London, London, UK; 3 CR UK & UCL CTC, NCRI, London, UK; 4 Edinburgh Clinical Trials Unit, The University of Edinburgh, Edinburgh, UK; 5 Mount Vernon Cancer Centre, Northwood, UK

**Keywords:** Data verification, quality, independent review, data entry, monitoring, error rate

## Abstract

**Background:**

The ICON6 trial (ISRCTN68510403) is a phase III academic-led, international, randomized, three-arm, double-blind, placebo-controlled trial of the addition of cediranib to chemotherapy in recurrent ovarian cancer. It investigated the use of placebo during chemotherapy and maintenance (arm A), cediranib alongside chemotherapy followed by placebo maintenance (arm B) and cediranib throughout both periods (arm C). Results of the primary comparison showed a meaningful gain in progression-free survival (time to progression or death from any cause) when comparing arm A (placebo) with arm C (cediranib). As a consequence of the positive results, AstraZeneca was engaged with the Medical Research Council trials unit to discuss regulatory submission using ICON6 as the single pivotal trial.

**Methods:**

A relatively limited level of on-site monitoring, single data entry and investigator’s local evaluation of progression were used on trial. In order to submit a license application, it was decided that (a) extensive retrospective source data verification of medical records against case report forms should be performed, (b) further quality control checks for accuracy of data entry should be performed and (c) blinded independent central review of images used to define progression should be undertaken. To assess the value of these extra activities, we summarize the impact on both efficacy and safety outcomes.

**Results:**

Data point changes were minimal; those key to the primary results had a 0.47% error rate (36/7686), and supporting data points had a 0.18% error rate (109/59,261). The impact of the source data verification and quality control processes were analyzed jointly. The conclusion drawn for the primary outcome measure of progression-free survival between arm A and arm C was unchanged. The log-rank test p-value changed only at the sixth decimal place, the hazard ratio does not change from 0.57 with the exception of a marginal change in its upper bound (0.74–0.73) and the median progression-free survival benefit from arm C remained at 2.4 months. Separately, the blinded independent central review of progression scans was performed as a sensitivity analysis. Estimates and p values varied slightly but overall demonstrated a difference in arms, which is consistent with the initial result. Some increases in toxicity were observed, though these were generally minor, with the exception of hypertension. However, none of these increases were systematically biased toward one arm.

**Conclusion:**

The conduct of this pragmatic, academic-sponsored trial was sufficient given the robustness of the results, shown by the results remaining largely unchanged following retrospective verification despite not being designed for use in a marketing authorization. The burden of such comprehensive retrospective effort required to ensure the results of ICON6 were acceptable to regulators is difficult to justify.

## Background

The extent of data verification conducted on a trial led by a pharmaceutical company with a marketing authorization in mind and one conducted more pragmatically with a focus principally on the advancement of knowledge of the intervention can diverge substantially. Usually this is a practical decision driven by its feasibility as extensive verification of data is costly with only limited value to academic sponsors.

The ICON6 trial (NCT00532194) is an academic-led, randomized phase III clinical trial investigating the use of cediranib (original tentative marketing name Recentin and later Zemfirza, made by AstraZeneca) in addition to a standard chemotherapy regimen in relapsed ovarian cancer. This UK Medical Research Council–sponsored trial was conducted pragmatically, allowing investigator-choice chemotherapy and delegating roles and responsibilities for conduct and regulation to each of the four international collaborative groups. These groups were the UK Medical Research Council-National Cancer Research Institute, Canadian Cancer Trials Group, Australia New Zealand Gynecological Oncology Group and Grupo Español de Investigación en Cáncer de Ovario. The trial was centrally coordinated by the Medical Research Council Clinical Trials Unit at University College London (the “Unit”). Centralized risk-based monitoring was used in a proactive manner throughout the conduct of ICON6 with, for example, consent and eligibility checks, database plausibility algorithms and triggered for-cause site visits (see full details in the Online Appendix). The trial had enrolled fewer than 390 patients (from a planned 2000) when drug supply availability drove a redesign and premature termination of recruitment.

**Figure 1.**
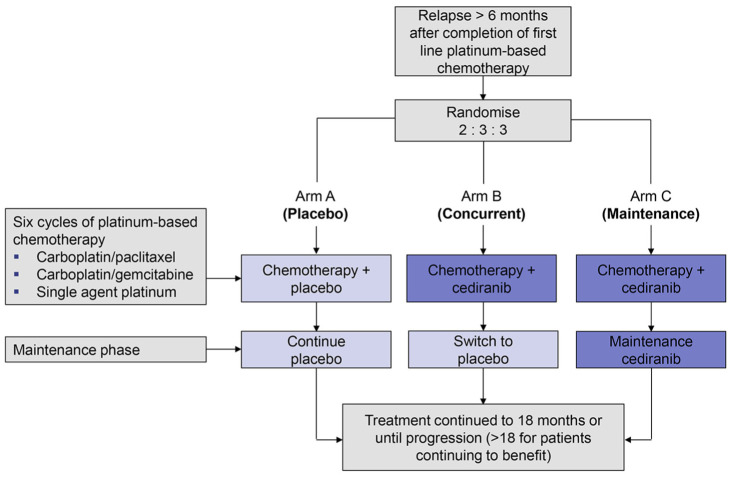
ICON6 trial design.

Patients were randomized to receive one of three arms: chemotherapy with placebo throughout (arm A); chemotherapy with concurrent cediranib, followed by placebo during a maintenance period (arm B); and chemotherapy with concurrent cediranib, followed by maintenance cediranib (arm C). This trial design is shown in Figure 1. The original pre-specified primary outcome measure was a comparison of the length of overall survival between arms A, B and C. Secondary outcome measures included progression-free survival (time to progression or death from any cause), toxicity and quality of life.

While ICON6 was still accruing patients, AstraZeneca discontinued manufacturing the drug, following evidence from other randomized clinical trials that cediranib was not effective in several tumor types (colorectal cancer, non-small-cell lung carcinoma and glioblastoma).^1–3^ This meant a reduction in the target sample size of ICON6 to approximately 470 patients owing to the very limited remaining supply of the drug, and a reconfiguration of the primary comparison to maintain as much power as possible with the much smaller sample size. The primary outcome was swapped from overall survival to the more frequent, and earlier occurring, measure of progression-free survival (overall survival is discussed in detail in the Online Appendix). The primary comparison was also revised from arms A versus B versus C to only compare arms A versus C which was considered the most appropriate comparison to target in view of emerging data for concurrent and maintenance bevacizumab. It is important to note that this change of primary outcome was made before any interim analysis was performed of efficacy outcome measures.

Results of the pre-specified primary analysis showed clear evidence of an improvement in progression-free survival with a hazard ratio of 0.57 (95% confidence interval 0.45–0.74; p = 0.0000097) and a 2.3 month improvement in the difference between the median time before the cancer returned (arm C vs arm A). This improvement from 8.7 to 11.1 months in the median time to progression or death from any cause would be considered by many to be a clinically meaningful improvement in relapsed ovarian cancer, a setting where patients receive diminishing absolute benefit from each subsequent treatment regimen post-progression and the duration of the response is short, although this benefit came with added toxic effects. These trial results were initially presented at the Presidential Session of the European Cancer Congress in 2013 and later, after the work described here, published in *The Lancet* in 2016.^4,5^

As a consequence of the results of ICON6, AstraZeneca restarted drug manufacturing and initiated preparation of a marketing authorization application, initially in the European Union with the European Medicines Agency (EMA), and also potentially in the United States with a submission to the Food and Drug Administration (FDA).^6^ The lack of benefit seen in other tumor types meant that, despite its limited size, ICON6 would be the single pivotal randomized phase III trial in the submission. It was to be supported by NCI 8348 that a randomized phase II trial in 90 patients demonstrates a progression-free survival improvement for cediranib plus olaparib over olaparib alone in the same setting of relapsed ovarian cancer.^7,8^ ICON6 had been conducted using a long-established risk-based monitoring approach that is common in clinical trials sponsored by our academic Unit and is consistent with recent approaches advocated by EMA/FDA.^9^ However, this is very different to typical company-sponsored monitoring practices and in any case, plans for on-site monitoring in ICON6 had been weighted toward triggered for-cause visits in the middle to later stages of the trial, so had been infrequent at the time recruitment was prematurely terminated. Consequently, the trial team in conjunction with AstraZeneca instigated a retrospective data verification process. Retrospective data verification was undertaken within three broad themes of source data verification, quality control checks and blinded independent central review of scans as it was judged that the regulator would require such processes. In this article, we report the impact of these verification processes on the key efficacy and safety results.

## Methods

After presentation of the primary results, the Unit and AstraZeneca agreed how ICON6 might be prepared to be the pivotal trial within a regulatory submission. Key areas, and our proposed actions, were as follows: (a) source data verification—checking of the completed paper case report forms against the source patient notes at site; (b) quality control checks—verification of the data entry of the paper forms onto the trials unit’s database; (c) blinded independent central review—independent reassessment of the patient’s locally held tumor scans to adjudicate the time point of progressive disease.

All analyses retained the same data freeze date of 19 April 2013, with only data prior to this cut-off contributing to the primary analysis. This revised database was used to assess changes in the primary outcome progression-free survival, and the four key toxicities emphasized in the initial presentation due to the nature of this class of drug.^4^

### Source data verification

Centralized monitoring based on set risk factors was planned to be used throughout the trial but supplemented with on-site visits of all UK sites at least once during the course of the trial in addition to any triggered visit. However, as the trial was shortened due to drug supply, this was not possible. Details of the monitoring plan, and updates to it, is given in the Online Appendix.

Checking visits for retrospective source data verification were fulfilled by a third-party contract research organization arranged by AstraZeneca across all countries over the space of 7 months. This typically involved two clinical research associates visiting each site for 1–2 days and totaling 594 visits. This required a large amount of coordination both at the Unit and at the individual sites, because sites had to retrieve the relevant patient notes and the completed trial forms prior to each visit. During source data verification, the form used was compared against the patient notes. Any discrepancies between the source patient hospital notes and the form were documented. These discrepancies were then resolved by the site via corrective action on a Data Clarification Form or the completion of a new form, which was then sent to the Unit for data entry.

This process of comparing the source notes and the form was completed for data critical to the reporting of the trial’s key outcome measures. These data included select fields of the screening, final safety visit, follow-up and progression forms, and all data on the tumor assessment, adverse event (AE), serious adverse event (SAE), end of trial drug summary and death forms. Further detail is given in the Online Appendix.

### Quality control checks

Quality control checks were performed to ensure that the data entered on to the Unit’s database accurately represented the completed form sent from the site. This process was deemed prudent as the Unit used trained single data entry as per local policy. The Unit’s policy of trained single data entry is based on the conclusion that when automatic database validation checks and statistical error checking are used, there is only a small marginal gain in accuracy from doubling of the data entry time and cost.^10,11^ Whereas for many company-sponsored studies double data entry would be used.

This second aspect of checking was initiated once the source data verification was completed with all queries resolved and closed, and any new forms entered onto the database. The data extracted was compared to a scanned copy of the paper form and any related documentation. A quality control error was defined as any difference between the form and the study database where there was no supporting documentation for the inconsistency. The error rate was calculated using the total number of valid, newly reported discrepancies divided by the total number of data points checked.

The process of checking the form against the database comprised two distinct components, performed in parallel: (a) *Key data*—data points critical to the primary efficacy analysis were examined for errors in all 486 patients and (b) *Supporting data*—data points contributing to the detailed reporting of the trial were examined for errors in 25 of 486 patients (5% sample).

Data points deemed to be *key data* by the Trial Management Team and so checked for all patients were as follows:

- Screening form—date of randomization
- Follow-up form—survival status, date last seen alive
- Progression form—date of progression
- Death form—date of death
- SAE form—main symptom, grade, date of onset, causal relationship to SAE, expectedness, action taken due to SAE

Data points deemed to be *supporting data* were more extensive and the same as reviewed in the source data verification, these are described further in the Online Appendix. The 5% sample of patients consisted of two patients from each of the five highest recruiting sites in the United Kingdom; plus 12 patients from the remainder of the UK sites (22 of 380 UK recruited patients); and one patient randomly selected from each of the non-UK collaborating nations, Canada, Australia and Spain, who recruited, respectively, 87, 17 and 2 patients. An initial 5% sample of patients, expanded if deemed necessary, was done to ease the burden on sites given the age of the trial and the retrospective nature of the verification.

It was decided that for *key data* errors, an error rate of >0.5% (a common threshold in company-sponsored trials)^12^ would signal issues with the trial conduct and for *supporting data* errors, it would warrant further investigation with checking promptly expanded into 100% of trial participants. All errors identified in the course of both quality control processes were corrected.

### Blinded independent central review ofprogression scans

Blinded independent central review was coordinated by AstraZeneca using a specialist contract research organization to assess the study imaging and to determine an overall cancer tumor assessment at each visit using RECIST (Response Evaluation Criteria in Solid Tumors) criteria.^13,14^ Slight differences were anticipated as reviewing scans is subjective to an extent, and within the trial investigators could also declare progression based on “general deterioration” alone.

Unlike source data verification and quality control, the blinded independent central review was a sensitivity analysis of the primary efficacy outcome (progression-free survival) rather than a refinement. The blinded independent central review analysis subset, limited to patients who had at least one scan or radiological report provided for review, was used to assess ascertainment bias and comprised 96% of the all patients randomized intention-to-treat population.

There was a minimum of six independent, qualified, board-eligible radiologists assigned to the evaluation. These reviewers assessed the study imaging to determine overall tumor assessment at each time point using RECIST criteria. Each patient’s data was reviewed blinded to treatment allocation by two reviewers using two methods: (a) primary review of each patient’s tumor assessment at each time point and (b) global review of all time points combined. If the two independent reviewers disagreed, a third reviewer adjudicated.

## Results

### Source data verification/quality control data point changes

The site visits for source data verification took place over 7 months, March–September 2014. In total, 594 individual Clinical Research Associate visit days were completed across all 63 recruiting sites. Source data verification was completed in full for 444 of 456 (97%) patients on all three arms. The remaining 12 patients’ (3%) notes were irretrievable, but almost all could be partially monitored using annotations, laboratory reports and scan records. A total of 3253 queries were raised, which is an average of 6.7 per patient. It took on average 1.2 days to complete the required checks for each of the 486 patients recruited to the trial. The contract research organization performing the source data verification did not share data point changes with the Unit in a summarized format, though the Unit did record them for the quality control.

Changes arising from the quality control were: (a) *Key data*—7686 data points were quality control checked and 36 errors were identified, which is an error rate of 0.47% and (b) *Supporting data*—25 of the total 486 patients were checked; 59,261 data points were quality control checked and 109 errors were identified, which is an error rate of 0.18%.

The *key data* quality control, in 100% of patients, discovered 30 errors related to SAE reporting (5% of 571 events), five to follow-up and one to death (0.4% of 225). The death-related error was a data entry error where the date of death was recorded as the date of form completion. The five errors related to the follow-up form were a variety of data entry errors, mainly a one-digit error in the date last seen entry. The most common errors of the 30 in the SAE forms were 10 errors in the action taken in regard to trial treatment in response to the SAE and eight related to the causal relationship of the trial treatment to the SAE. The others related to expectedness (four), date of onset (three), SAE grade (three) and SAE name (two). No expedited reporting of serious adverse reactions to sponsor or regulators was affected.

In terms of the *supporting data* quality control, in a 5% sample of patients, 109 errors were detected in 59,261 checked data points (0.18%). The vast majority of these errors (78%, 85/109) were related to reporting of AEs. 29% of the errors were misreported grade of severity. These errors are concerning given the critical nature of safety reporting, though with up to 71 grades to be entered on a single extensive AE form; over numerous visits at baseline, chemotherapy and follow-up phases; and with 25 patients selected for review this error rate is extremely low in totality.

### Source data verification/quality control efficacy impact

It is not feasible to isolate and assess the efficacy impact of the source data verification and the quality control separately; consequently, the results of these two processes will be presented together. Full statistics are provided in Table 1.

**Table 1.** Efficacy results for the primary outcome (progression-free survival, PFS) (a) as initially presented, (b) following the source data verification (SDV) and quality checking (QC) processes and (c) following the blinded independent central review (BICR).

	Initial presentation		Following SDV/QC		Following BICR	
	Arm A	Arm C	Arm A	Arm C	Arm A	Arm C
Population	Intention-to-treat		Intention-to-treat		BICR subset	
N^a^	118	164	118	164	113	158
PFS events^b^ (%)	112 (95%)	139 (84%)	113 (96%)	140 (85%)	102 (90%)	128 (81%)
Log-rank test	p = 0.0000097		p = 0.0000075		p = 0.00074	
Grambsch–Therneau test	p = 0.024		p = 0.062		p = 0.065	
Hazard ratio (95% CI)	0.57 (0.45–0.74)		0.57 (0.44–0.73)		0.64 (0.49–0.83)	
Median PFS in months (95% CI)	8.7 (7.72—9.49)	11.1 (10.38—11.96)	8.7 (7.72—9.40)	11.0 (10.38—11.73)	9.4 (8.31—10.09)	11.1 (10.68—11.99)
Restricted mean survival time(RMST) in months (95% CI)	9.6 (8.49—10.32)	12.7 (11.63—13.48)	9.4 (8.60—10.19)	12.5 (11.69—13.36)	10.6 (9.61—11.57)	13.4 (12.49—14.33)

CI: confidence interval.

a 174 patients were randomized to arm B which, along with the 118 in arm A and 164 in arm C, totaled 456 patients. Another 30 patients were initially randomized between the three arms at the 30 mg dose, before the trial was restarted at 20 mg, totaling 486 patients.

b A PFS event is defined as progression or death from any cause.

After source data verification and quality control, two previously unreported progression events were identified: one on each arm (A and C), resulting in a 1% increase in event rate in each arm. This actually represented the observation of three more progression events prior to the date of data cut-off, but one progression event was recorded in error pre-source data verification because the patient had asked for no further follow-up on the trial. Overall, 34 changes to event time were made, 11 in arm A and 23 in arm B (2:3 randomization ratio), with a median change of −7 days for arm A and −3 days for arm C.

The log-rank test for equality of the curves provided very strong evidence of a difference between arm A and arm C both initially and after source data verification/quality control, with a change only at the sixth decimal place.

For the hazard ratio, there was only a minor adjustment on one side of the confidence interval. The restricted mean survival time estimated gain remained at 3.1 months with small adjustments in the estimates of the two arms. In the statistical analysis plan, it was stipulated that if there was evidence of non-proportionality, the restricted mean survival time would be used as the primary point estimate of the treatment effect, and the hazard ratio if there was no evidence, though both estimates would reported as a supportive evidence.^15^ The Grambsch–Therneau test for non-proportionality p-value change did result in an adjustment in interpretation, and so consequently the primary description of the difference became the hazard ratio over the restricted mean survival time.

Median progression-free survival times shortened slightly in arm C but remained with a 2.4 month gain in time to progression or death.

The conclusion drawn for the primary outcome measure of progression-free survival and in the primary comparison of arm A versus arm C was therefore unchanged from the initial presentation.

**Figure 2.**
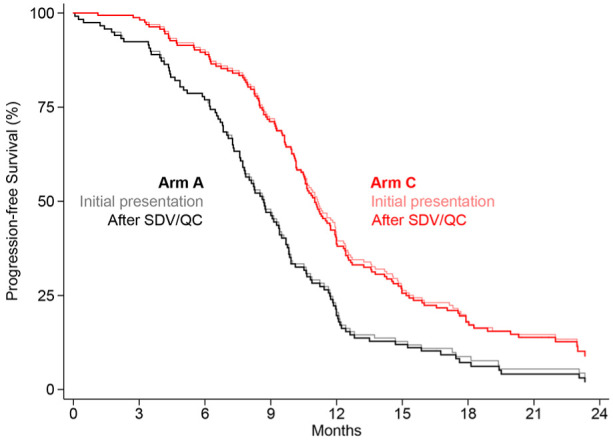
Kaplan–Meier PFS plot displaying estimates of (a) initial presented results and (b) SDV/QC overlaid.

In Figure 2, the primary visual comparison of arm A versus arm C as a Kaplan–Meier plot is presented, clearly illustrating the marginal effect on the primary efficacy endpoint following the source data verification and quality control processes.

### Blinded independent central review of scans

The number of patients included in the analysis set in the blinded independent central review was reduced by 4% in each arm in comparison with the local evaluation, due to only including those patients with at least one scan or radiological report provided for review. This meant the proportion of events was reduced in absolute terms by 5% in arm A and by 3% in arm C. In arm A, one patient (1%) had a newly identified progression and eight patients (7%) were classed as not having progressed—contrary to the primary analysis. The pattern in arm C was rather similar in that two patients (1%) had progressions added and 13 (8%) had theirs omitted. Overall, the concordance rate in ICON6 between the local evaluation and the blinded independent central review–based analyses was 19%, which is comparable with other contemporary trials that have reported their concordance such as AGO-OVAR 16 (15%), GOG-0218 (23%), OCEANS (26%) and AURELIA (31%).^16–19^ Again, all statistics are presented in Table 1.

**Figure 3.**
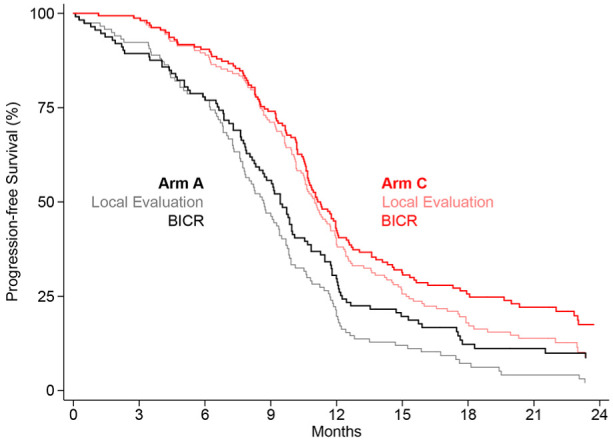
Kaplan–Meier PFS plot displaying estimates of (a) local evaluation (LE) and (b) blinded independent central review (BICR) overlaid.

The strength of evidence for a progression-free survival difference between the treatment arms using the log-rank test in the blinded independent central review analysis decreased slightly, though still provided very strong evidence of a difference and should not be a surprise given the decrease in both population size and event rate. This difference in survival curves is presented graphically in Figure 3. The drop in the number of events in both arms and consequently the lift in the curves in each arm is clear, but the overall consistency of the difference in treatments over time is comparable (described in the Online Appendix). The test for non-proportionality p-value was different to the initial presentation and very similar to the post–source data verification/quality control data, consequently the hazard ratio was used as the primary estimate of the difference. The size of the hazard ratio benefit of arm C was similar in both the local evaluation and blinded independent central review, but marginally stronger in the local evaluation. The restricted mean survival time for the blinded independent central review–based analysis followed the same pattern of a very slight reduction in the estimated gain.

### Toxicity reporting impact

Toxicity on the trial was collected using Common Terminology Criteria for Adverse Events (CTCAE) from the National Cancer Institute.^20^ Grades within this system broadly correspond to the following: Grade 1 “Mild”; Grade 2 “Moderate”; Grade 3 “Severe or medically significant but not immediately life-threatening”; Grade 4 “Life-threatening consequences”; and Grade 5 “Death related to AE.”

During the conduct of ICON6, the National Cancer Institute revised the CTCAE scales, updating from version 3 to version 4. This occurred on 28 May 2009 by which time 71 of the eventual 486 patients had been recruited. The guidance for sites participating in the trial was that patients should continue with the same version of the form (and consequently same version of the AE scale) with which they started on the trial to enable comparison with baseline grades given the change in some key toxicities definitions. This meant 15% (71/486) should have assessed patients using version 3, with the rest using version 4.

**Figure 4.**
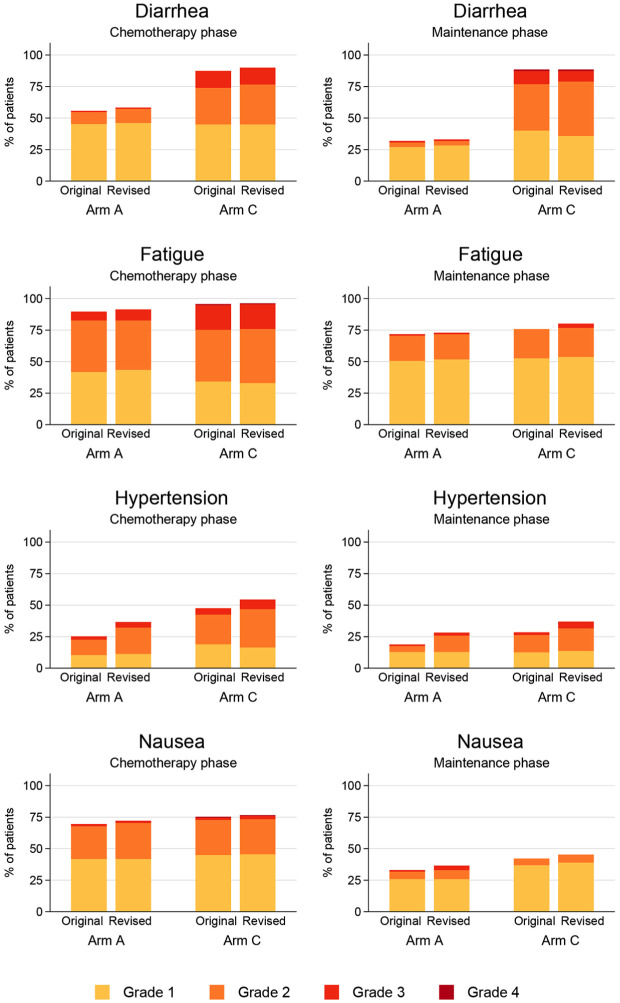
Maximum adverse event grade (CTCAE) experienced, before and after SDV/QC, by trial phase—using the safety analysis set for the chemotherapy phase (A = 115/118, C = 158/164) and the maintenance phase (A = 85/118, C = 95/164).

The four key toxicities associated with vascular endothelial growth factor treatments and highlighted in the primary presentation are given in Figure 4.^4^ This figure displays the highest grade of each toxicity experienced on trial, separately for the two phases (during chemotherapy and on maintenance). As shown by the left-hand four plots, as expected, patients report very high levels of all four toxicities during the chemotherapy phase. Comparing the “original” and “revised” bars, after source data verification/quality control, there were only very small uplifts in maximum grade reported in diarrhea, nausea and fatigue—but some important differences within hypertension. This pattern of a lack of difference for diarrhea, nausea and fatigue but increase in hypertension was also observed in the maintenance phase, as shown by the right-hand four plots, and so warrants further discussion. Within both the chemotherapy and maintenance phases, 12% of patients had their maximum hypertension grade altered and all of these changes were uplifts in grade. The majority of the hypertension changes were due to blood pressure readings in the source notes (70%) or the start of anti-hypertensive treatment (17%) which had not been reported on the forms for the trial. There was no evidence of a systematic bias by arm.

We had informal discussions with investigators around the reasons for blood pressure readings not being recorded on forms. The conclusion being that this was a specific circumstance where the definition had changed and staff were more familiar with the older scale classification from version 3, substantially based on need for changing therapeutic regimens, whereas the updated version 4 mandated a change in grade based on firm numerical guidelines. Many clinicians would have been comfortable with the older system and graded according to it. For example, a patient observed in clinic with a one-off blood pressure reading of systolic 140 or diastolic 90 previously would have been ungraded, but in the revised version would have been assigned grade 2 hypertension. The consequence being that some elevated blood pressure readings that met version 4 criteria were not always recorded as hypertension AEs, but Clinical Research Associates completing the source data verification identified these as additional AEs. This may have been particularly so when the clinician considered the blood pressure reading was likely to be related to stress in clinic.

In the original trial report, AEs were formally compared if they were experienced by >5% of the trial population (described in the preceding paragraph), or if they were high grade (“life-threatening” 4 or “fatal” 5) of any frequency. Within this high-grade AE reporting group, only two occurrences were altered. For one patient, their pneumonia was revised from grade 4 to grade 5 (arm A), and one grade 3 pancreatitis revised to grade 5 (arm C). Both had been appropriately reported as fatal SAEs, and given the difficult nature of assigning a definitive cause of death to a specific symptom, minor changes are not surprising. There was a downgrading from grade 4 to grade 3 hypomagnesemia for one arm C patient.

## Conclusion

We found that the three themes of retrospective data verification (source data verification, quality control and blinded independent central review) made only immaterial changes to the key outcome measures of the trial—and consequently its interpretation.

The two most time-consuming parts, the source data verification and quality control, had the least impact on the primary efficacy outcome measure. The impact of source data verification was consistent with previously reported experiences, one of which states, “the true effectiveness value of source data verification […] is minimal (0.1%–0.4%)”^21^ and another which states that “discrepancies identified made no impact on the main conclusions of the study.”^22^ The quality control process did discover errors, in the *key data* this almost met the 0.5% threshold we set that would signal concern with trial conduct, but overall compared well to error rates in the literature and again made no impact on the interpretation of the study.^23^ The blinded independent central review sensitivity analysis, on the contrary, did make a difference to individual assessments, as was expected given the complexity and partially subjective nature of declaring progression in cancer patients, especially retrospectively. This sensitivity analysis supported the primary, local evaluation, result in that the overall test of a difference and the size of the estimate remained overwhelmingly positive. Consequently, the local evaluation conclusion that cediranib increases time to progression or death was reinforced. The literature contains much discussion as to the limited added value of blinded independent central review already and suggests that local evaluation “provides a reliable estimate of treatment effect” in most settings in the study of cancer.^24,25^

As anticipated, there were some changes in the toxicity profile. For the most part, these were minor and made little or no difference to the standard toxicity summary statistics and do not appear to have been systematically biased toward one treatment arm. Some additional hypertension episodes were reported, though in discussion with treating gynecological oncologists we feel that this is likely due to substantial differences in the method of grading hypertension between versions of the AE system used. This meant that what many interpreted to be one-off, non-clinically significant blood pressure readings were graded as a low-grade AE in the updated AE scale—for which a subset of investigators had not realized had been revised. This substantial difference in definition of hypertension between versions is emphasized by Akhtar et al. who, in study NCI 6981, observed the majority of patients (65%) increased by two grades in their retrospective regrading using version 4. They concluded the impact of this change as “particularly important with vascular endothelial growth factor-targeted agents” such as cediranib.^26^ It is our belief that these changes would not have altered the overall toxicity assessment as the changes were not biased toward one arm, it was already acknowledged that the toxic effects of vascular endothelial growth factor inhibitors can be problematic, and prompt management guidelines were generally successful in controlling further hypertension.

A risk-based monitoring approach was used in the ICON6 trial, as is standard at the Unit given studies have demonstrated it to be non-inferior to extensive on-site monitoring.^27^ The EMA and FDA published their support for alternative approaches such as centralized, risk-based monitoring in 2013 but the practice of extensive on-site source data verification was, and is still likely to be, commonplace in company-sponsored trials.^9,28,29^

This retrospectively performed work took substantial amounts of time. Our crude estimate is that it was the primary task for our trial team for 2 years, equivalent to 4.5 full-time staff members per year. External contractors were used for more focused periods, but we estimate this to be equivalent to nine full-time staff per year. It is likely that retrospectively conducting these processes would be more time-consuming than if done prospectively.

The conduct of this pragmatic, academic-led trial was sufficient given the robustness of the results, shown by the results remaining largely unchanged following retrospective verification. The burden of such comprehensive retrospective effort required to ensure the results of ICON6 were acceptable to regulators is difficult to justify.

## Supplemental Material

Appendix_rev3_clean – Supplemental material for Impact of retrospective data verification to prepare the ICON6 trial for use in a marketing authorization application Supplemental material, Appendix_rev3_clean for Impact of retrospective data verification to prepare the ICON6 trial for use in a marketing authorization application by Andrew Embleton-Thirsk, Elizabeth Deane, Stephen Townsend, Laura Farrelly, Babasola Popoola, Judith Parker, Gordon Rustin, Matthew Sydes, Mahesh Parmar, Jonathan Ledermann and Richard Kaplan in Clinical Trials
