# Supplementary material for: Impact of retrospective data verification to prepare the ICON6 trial for use in a marketing authorization application
Source: Clin Trials. 2019 Jul 26;16(5):502–11. doi: 10.1177/1740774519862528 (PMC6801797; doi:10.1177/1740774519862528)
Supplement: Appendix_rev3_clean – Supplemental material for Impact of retrospective data verification to prepare the ICON6 trial for use in a marketing authorization application [file Appendix_rev3_clean.docx]

Appendix

Relevant passages of the ICON6 Monitoring Plan and its revisions

| **Version** | **Date** | **Detail** |
| --- | --- | --- |
| **1.0** | **03-Jun-2008** | **This document primarily describes the monitoring plan for stage I of the three stage design.**  **As described in the stage I analysis 'The primary aim for stage I was to determine the safety and feasibility of adding cediranib to platinum-based chemotherapy. If concurrent cediranib and chemotherapy were found to be tolerable at the stage I analysis, then the trial would be expanded and proceed to stage II. The primary outcome measures for stage II is activity as assessed by effect on progression-free survival (PFS), and for stage III it is overall survival (OS). The aim is to recruit 2000 patients for the third-stage analysis.' [*British Journal of Cancer* volume 105, pages 884–889 (27 September 2011)]**  *Monitoring programme*  Following a risk assessment for this trial, the TMG have agreed that monitoring will take the form of central monitoring procedures with triggered on-site monitoring.  *Initiation visits*  Initiation visits will not be formally performed for all sites. A training package of start-up slides is available and will be updated regularly. Investigators who have not attended the launch meeting will be asked to review the slides. Any questions that arise from the sites will be addressed by email or phone calls with either the Trial Manager, Trial Physician or Chief Investigator. A visit to the site will be arranged if it is felt to be useful to discuss any further issues.  *Central monitoring*  Stage I monitoring will be conducted centrally unless one of the triggers below suggests a site monitoring visit is necessary.  The MRC CTU staff will:   - perform data entry (database) plausibility checks for validity and consistency of data; - identify missing or inconsistent data; - review consent forms; - identify and discuss random or systematic errors feasible during the course of data collection; - identify protocol violations; - check that CRFs are completed by authorised persons against the signature and delegation log; - review recruitment rates; - Provide centres with self-assessment check sheets for essential documentation to verify the correct versions are in use; - Remind centres regularly of CRF completion timelines and SAE procedures; - Annually request updates of the signature and delegation log, and the personnel list; - Arrange self-certification of key data points from source data to be checked against data received at MRC CTU   The trial physician will   - perform a manual check of CRFs for all patients in stage I for data consistency - document whether or not clinical guidelines were followed for management of hypertension, diarrhoea, proteinuria and renal impairment   *Completion*  At the end of stage I a review of the trial data will inform a revised monitoring schedule for subsequent trial stages.  *Visit triggers*  Triggers that would indicate a monitoring visit is required include, but are not restricted to, the following:   - Poor recruitment - Over or under reporting of SAEs - Unusually low return of consent forms - Unusually low reported event rate - Large amount of missing data - Large amount of data queries - Concern by the trial physician or trial management staff raised by the central review detailed above. - Inappropriate drug administration. (Study drug discontinuations, dose modifications and drug interruptions will be flagged on the database and other clinical data points reviewed for appropriateness of this drug modification. The first time study drug administration is inappropriately followed, the trial physician/clinical deputy/CI will contact the site to discuss the case and the sites’ understanding of the procedures. If inappropriate drug administration is noted a subsequent time at the same site a monitoring visit will be triggered.)   These will be reviewed by the TMG on at least a 3 monthly basis.  *At the visit*  Checks that the centre is conducting the trial in accordance to ICH-GCP, and Source Data Validation on the patient data will be performed:   - Key eligibility variables will be source data checked where possible - SAR/SUSAR and Notable Adverse Reaction reports will be source verified against clinical notes - Data from patients experiencing study drug discontinuations and interruptions will be reviewed - If <5 patients have been randomised at the site, all patient files will be checked for unreported grade 3 and 4 toxicities (If >5 patients have been randomised a random 5 patient files will be checked) - Patients with disease progression will have files monitored for RECIST criteria to make an assessment of CRF design, and to check that procedures and guidance are appropriate for their use in later trial stages - Trial drug dispensing, destruction and drug supply inventory will be checked for completeness.   *Patient Source Data Verification*  For each patient to be monitored, the following data need to be validated:   - Consent for the trial - Eligibility for the trial (including lab values) - Maximum grade of each toxicity reported during a follow up period - Details and follow-up of any serious adverse drug reaction(s) - Disease progression and date(s) of progression - Date and cause of death - Unreported events |
| **2.0** | **10-Aug-2010** | **This document focusses on plans for efficacy stages II—III. Stage I was completed in Nov-2009, with an acceptable safety profile.**  *Organisation*  Updated to state that monitoring may be performed by the relevant GCIG collaborating group, and that AZ staff may accompany MRC staff at site visits.  *Central monitoring*  The plan remained central monitoring with triggered visits when necessary – though updated to state each site in the UK would be visited at least once during the lifetime of the trial, with highest recruiters (>30 patients) and 'for-cause' triggered visits occurring first. Sites coordinated by the collaborating GCIG groups may follow their own national monitoring plan following consultation with the Trial Management Group.  *Completion*  At the end of stage II a review of the trial data may inform a revised monitoring schedule for the next trial stage.  *Visit triggers*  Only addition to the visit triggers being the addition of 'Sites that do not return CRFs or queries within the timelines defined in the Data Management Plan'  *Patient Source Data Verification*  Patient SDV remained the same.  *GCIG monitoring*  This new section broadly describes the approaches GCIG groups should take in their monitoring approaches.  Stating this can be in the form of:   - Central monitoring - Annual meetings for trial review - Sites completing an initiation questionnaire detailing their responsibilities - Issues identified for discussion at team meetings - Collection of GCLP certification and lab accreditation documents - Regular recruitment review |
| **3.0** | **24-Feb-2014** | **This monitoring plan follows the cessation of cediranib manufacture as the subsequent redesign in Sep-2011 and the completion of recruitment in Dec-2011.** **It also follows the presentation of the primary (PFS) results in Sep-2013.**  *Overall*  Some minor wording changes to reflect the fact the recruitment had completed.  *Central monitoring*  A clarification of the monitoring strategy was added:  Stage II monitoring is also conducted centrally, although it was the intention in stage II to visit all UK sites at least once during the lifetime of the trial even if a ‘for cause’ visit had not been triggered, with high recruiters (>30 patients) being monitored first. However, due to the early closure of recruitment in stage II (the trial will not progress into stage III) it is no longer possible to visit each site in the UK due to time constraints. Therefore it is likely that only 'for cause' visits will take place.  The two following sections were added to reflect the licence submission and the retrospective visits described in this manuscript:  *On-site monitoring visit (AZ/CRO co-ordinated)*  The visits that are taking place between Feb-June 2014 are co-ordinated by AZ/CRO in the run up to the licence submission. AZ will supply a separate communication plan which will give details of these visits and the data being verified/collected. All completed monitoring reports and DCFs will be sent to MRC CTU.  *Cediranib license submission to FDA and EMA*  The results of the ICON6 PFS analysis will be used for AstraZeneca to make a license submission for cediranib to both the FDA and EMA. The licence submission will involve a blinded independent central review (BICR) of all patient scans, and 100% source data verification of all critical data points (inclusion/exclusion criteria, progressions, deaths and safety data). AstraZeneca will contract out this work to Clinical Research Organisations (CRO’s). The CRO’s will be responsible for providing MRC CTU with a separate plan for the additional monitoring required for the cediranib license submission for review. |
| **4.0** | **06-May-2015** | **Annual review – no major changes** |
| **5.0** | **12-Jun-2017** | **Annual review – no major changes** |

Fields checked in Source Data Verification and *supporting data* Quality Checking

- Screening form – key inclusion/exclusion criteria, date of informed consent, date of randomization – totaling 36 variables
- Tumor Assessment form – all data – totaling 41 variables
- Adverse Event (AE) form – all data – totaling 249 variables
- Serious Adverse Event (SAE) form – all data – totaling 40 variables
- Final Safety Visit form – AE data – totaling 88 variables
- End of Trial Drug Summary form – all data – totaling 19 variables
- Follow Up form – survival status, date last seen alive, progression status – totaling 3 variables
- Progression form – date of progression, target lesion status at randomization, progression confirmation method, site of progression – totaling 5 variables
- Death form – all data – totaling 3 variables

Comparison of the smoothed restricted mean survival time difference over time, between arms A and C, by Local Evaluation and Blinded Independent Central Review


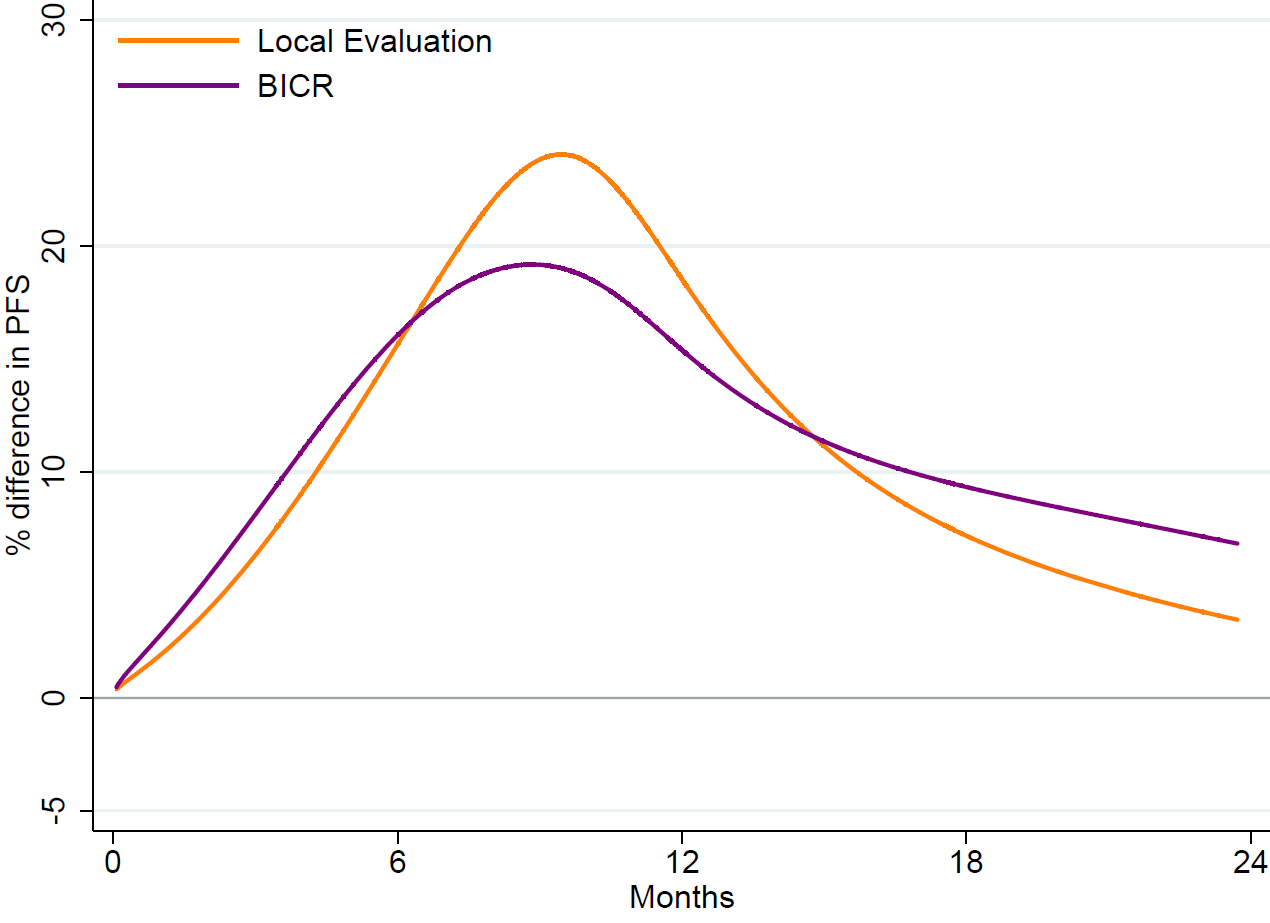


Overall Survival

One of the secondary objectives in ICON6 was the assessment of the effect of cediranib on Overall Survival. In the original, much larger, design Overall Survival was the primary outcome measure but this had to be revised to Progression-Free Survival in order for there to be sufficient numbers of events (progression or death) given the much reduced sample size. The reasons driving this revision are described fully in the primary ICON6 manuscript in *The Lancet* and in the main body of this manuscript.

At the time of initial presentation, in the Presidential Session of the European Cancer Congress 2013, the Overall Survival results were given as follows:

|  | **Arm A** | **Arm C** |
| --- | --- | --- |
| Deaths*, n (%) | 63 (53.3) | 75 (45.7) |
| Median, months | 20.3 | 26.3 |
| Log-rank test | p=0.042 | |
| Hazard Ratio (95% CI) | 0.70 (0.51—0.99) | |
| Test for non-proportionality p=0.0042 | | |
| Restricted means, months | 17.6 | 20.3 |

* death from any cause

These results were described as being ‘immature’ and presented without targeted chasing of survival status (or ‘survival sweep’) having been performed, this was due to the focus on the primary fully powered outcome of Progression-Free Survival.

Following the initial presentation and AstraZeneca’s decision to submit for licensing authorization for cediranib in Europe the decision was taken to replace a patient’s date of censoring with the data cutoff date (19-Apr-2013) under certain circumstances. Rather than using the date of the last patient visit prior to data cutoff, the revised approach brought censoring forward to the cutoff date for any patients who were confirmed alive following 19-Apr-2013 on the basis of receipt of one of the following forms: follow up, SAE, or death. This approach was undertaken in order to achieve the effect of a ‘survival sweep’ as would have been performed if Overall Survival had been the primary endpoint, and did produce some numerical differences between the Overall Survival results presented at the European Cancer Congress 2013 and the primary trial report/this manuscript.

After including extra deaths observed in both arms post-presentation dated before the cutoff date and incorporating the extra follow up from the censoring rule, the Overall Survival results were as follows:

|  | **Arm A** | **Arm C** |
| --- | --- | --- |
| Deaths*, n (%) | 64 (54.2) | 81 (49.4) |
| Median, months | 21.0 | 26.3 |
| Log-rank test | p=0.11 | |
| Hazard Ratio (95% CI) | 0.77 (0.55—1.07) | |
| Test for non-proportionality p=0.001 | | |
| Restricted means, months | 22.8 | 25.8 |

* death from any cause

50 patients in arm A and 74 in the maintenance arm had their censoring date brought forward to the date of cutoff (19-Apr-2013); given the 2:3:3 ratio of randomization this was not unexpected. The most striking differences were in the median change in days, as arm A gained an average of 93 days (Inter Quartile Range: 36—130) whereas arm C gained 48 days (21—95). It is difficult to say whether a three months increase in follow up versus a month and a half in the presented results suggests that arm A patients had been less closely followed and it cannot be excluded that this is due to a less stringent follow up in the control arm. Given the blinded nature of the trial we feel this would more likely be a random finding, which however may have contributed to the observed narrowing of the Overall Survival difference between the two arms when compared to the presentation. Also, there was the addition of 4% more deaths in absolute terms in arm C and 1% in arm A that pertained to prior to the cut off but had not been reported at the time of presentation.

Most noticeable was the change in the log-rank test of a difference between arms A and C, which was revised from p=0.042 to p=0.11. While modern statistical thinking has moved on from cutoff-based interpretations many would still see this as a change from a ‘significant’ result at the 5% significance level to a ‘non-significant’ outcome.

Consequently this change in interim, not yet mature results for the secondary outcome of Overall Survival may alter the interpretation of the trial for some individuals.
